# 

**Published:** 1897-05-15

**Authors:** 


**CADBURY'S COCOA**
"The standard of highest purity at present attainable."—*Lancet.*

**CADBURY'S COCOA**

NO ALKALIES USED.

### THE HOSPITAL

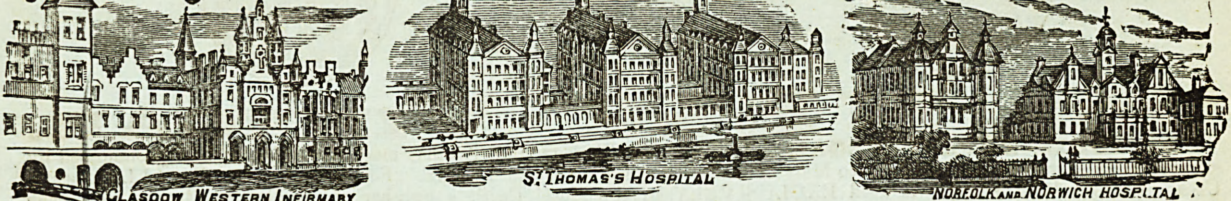

No. 555. Vol. XXII. [Registered as a Newspaper.] SATURDAY, MAY 15TH, 1897. [Subscription Terms, see p. 108.] **PRICE TWOPENCE.**

